# Prevention strategies for Alzheimer’s disease

**DOI:** 10.1186/2047-9158-1-13

**Published:** 2012-06-28

**Authors:** Serge Gauthier, Liyong Wu, Pedro Rosa-Neto, Jianping Jia

**Affiliations:** 1 McGill Center for studies in Aging, McGill University, Montreal, Canada; 2 Neurological Department, Xuanwu Hospital, Capital University, Beijing, China; 3 McGill Centre for Studies in Aging (MCSA), McGill University, 6825, Boul. LaSalle Blvd, Montreal, QC H4H 1R3, Canada

**Keywords:** Alzheimer disease, Risk factor, Prevention, Clinical trial, Clinical practice

## Abstract

Symptomatic treatment during the dementia stage of Alzheimer’s disease(AD) cannot delay or halt the progression of this disease. Therefore, prevention in the preclinical stage is likely the most effective way to decrease the incidence of this age-associated neurodegenerative condition, and its associated burden for individuals and society. Age, gender, family history, ApoE4, systolic blood pressure, body mass index, total cholesterol level and physical activity are all used as component of dementia risk score. There have been numerous challenges in conducting primary prevention trials in AD. Enrichment strategies for prevention studies include studying those subjects with more risk factors for AD, such as older age, those with a positive family history of late onset AD, and those who are ApoE4 positive. Each of these strategies is designed to increase the probability of developing AD thereby decreasing the sample size or the duration of follow up. Another strategy would be to target directly the pathophysiology of AD in its preclinical stages and use the biomarkers in prevention trial as surrogate markers. This will be done first in carriers of dominantly inherited early onset AD. As this research takes place networks of memory clinics must prepare to transfer new knowledge to persons interested in a preventive approach to AD.

## Introduction

Alzheimer disease (AD) is characterized by accumulation of amyloid plaques, neurofibrillary tangles and neuronal depletion associated with progressive deterioration of cognition and functional status [1]. AD is a catastrophic disease and symptomatic treatment (e.g. donepezil, rivastigmine, galantamine, memantine) during the different stages of dementia can only mildly ameliorate the symptoms and cannot delay or halt the progression of this disease, since extensive brain damage has already occurred prior to the dementia phase of AD [2]. Therefore, prevention in the preclinical stage is likely the most effective way to decrease the incidence of this age-associated neurodegenerative condition, and its associated burden for individuals and society [3]. There is great interest in prevention studies as a way to reduce the incidence and prevalence of dementias. This review will summarize the results of recent researches and outline some prevention strategies of AD for future research.

## Risk factors of AD

Numerous risk factors for AD have been identified by epidemiologic studies [4,5]. Everyone is at risk if living long enough (33% of individuals have AD over age 85), but some persons are more at risk than others because of their family history (Table 1). Family history in first-degree relatives is the main factor, and the age of onset of the family member matters as well: apoE4 genotype is more likely to be a factor if one of parent had AD at age 70 rather than at age 85 [6] .

**Table 1:** Proposed gradation of risk for AD in asymptomatic persons

• Basic risk (age)	
• Vascular risk factors	
• Family history of AD in first degree relatives but of late onset (>75)	
• Family history of younger onset (65–75)	
• Family history and ApoE4 +	
• Biomarker + (ex. PET amyloid positive scan)	
• Autosomal dominant mutation carrier	

Other known risks include subjective cognitive complaints [7] and demonstrable decline on serial cognitive testing even if still within the normal range considering age and education [8]. Another approach has been the assessment of a variety of risk factors in mid-life, giving them relative weights, and adding them up in a “Dementia Risk Score” [5], as summarized in (Mid-life dementia risk score [modified from 5]).

### Mid-life dementia risk score [modified from 5]

· Age at time of initial assessment

· Formal education level

· Gender

· Systolic blood pressure

· Body Mass Index

· Total cholesterol level in blood

· Level of physical activity

The new factors in the risk assessment towards AD are biomarkers: amyloid deposition evaluated by amyloid PET imaging and/or a reduction in levels of Aß42 in the cerebrospinal fluid (CSF), and neurodegeneration demonstrated by CSF, functional and structural imaging (e.g. tau of CSF, [18 F]-fluorodeoxyglucose positron emission tomography (FDG-PET) and structural MRI) [9]. The relative weight of these risk factors is still unknown, but at least 33% of cognitively normal persons over age 65 are “biomarker positive”. A new diagnostic category has been proposed by a National Institute on Aging (NIA) task force for such individuals, as summarized in Table 2[10].

**Table 2:** Asymptomatic persons with positive biomarkers of AD [modified from 10]

**Stage**	**Biomarkers or evidence**		
	**Aß (amyloid PET or CSF)**	**Neuronal injury (tau in CSF, FDG-PET, structural MRI)**	**Evidence of cognitive decline**
**asymptomatic cerebral amyloidosis (ACA)**	positive	negative	negative
**ACA + neuronal injury (NI)**	positive	positive	negative
**ACA + NI + subtle cognitive decline**	positive	positive	positive

## Prevention of AD

There have been numerous difficulties in conducting primary prevention trials in AD because of the unclear pathophysiological mechanism of AD, the difficulty in accurate selection of the target population, the need for a large sample size, long duration of follow up, the high cost of the prevention study, adverse events of the prevention drugs being studied and the related ethical issues [11-15]. Who should be enrolled in the primary prevention trials remains a very important but complex issue. The target populations of primary prevention are usually the healthy elderly. The subjects enrichment strategies include studying those subjects with more risk factors for AD, such as older people, those with a positive family history of AD, and those who are Apo E4 positive [11]. Each of these strategies is designed to increase the probability of developing AD thereby decreasing the sample size or the duration of follow up [11,16]. Another group of interest are persons with memory complaints but no measurable cognitive impairment (subjective cognitive impairment or “pre-MCI”), at higher risk of progression to dementia [17].

### Non-pharmacological interventions

Non-pharmacological interventions are possible: life style changes are of great interest to modify risk factors (predominantly vascular) and enhance protective factors (predominantly physical exercises, cognitive stimulation, healthy diet). Ongoing studies such as the the Multidomain Alzheimer Prevention Trial (MAPT) in Toulouse, France, combine omega-3 supplements with multi-domain interventions [18].

### Pharmacological interventions

Different risk and protective factors have been associated with AD, particularly in mid-life, and are amenable to a preventive approach [5]. Some prospective randomized studies targeting vascular risk factors such as systolic hypertension have demonstrated a reduction in the prevalence of dementia [19] but others not [20]. These equivocal findings may be explained in part by the variable effects of antihypertive drugs on pathways related to AD, but they have lead to a lack of endorsement by a US task force for the control of systolic hypertension as well as for any other life-style change as a preventive approach to AD [21] Nevertheless the weight of the evidence is that prevention of stroke is a good strategy to prevent dementia, either by control of vascular risk factors or by optimal post-stroke management.

Another strategy would be to target directly the pathophysiology of AD in its preclinical stages [22]. There are many possible pathophysiological targets for primary prevention in AD, including amyloid plaques, soluble amyloid, neurofibrillary tangles, loss of neurotransmitters, synapse loss, inflammation, and oxidative stress [11]. Table 3 lists some of these pathophysiological factors and potential drug treatments [2].

**Table 3:** Pathophysiology of AD and potential drug treatments

**Pathophysiology**	**Potential drug treatments**
amyloid deposition	beta and gamma secretase inhibitors active and passive immunotherapy
tau hyperphosphorylation	methylene blue, lithium, memantine
microglial activation	naproxen
inadequate synaptic plasticity	probuchol

Biomarker positivity can be important in deciding what population to enroll in a prevention study: more risk of progression to dementia will shorten the study but will limit the applicability of findings to the population as a whole [15,22]. Diagnostic biomarkers play an important role in population enrichment by refining selection criteria, stratifying populations and increasing the statistical power of trials. Endpoint biomarkers may be used as outcome measures to monitor the rate of disease progression and detect treatment effects of drugs [23].

The side effects of drugs are not negligible, particularly in asymptomatic persons. These risk/benefit considerations are very important to research ethics boards and regulators: “safety must be the primary consideration since an agent that will be administered to thousands of healthy normal individuals, many of whom will never develop disease, must be remarkably free of side effects” [24].

## Aims in prevention studies for AD

### Delaying biomarkers changes

The Alzheimer Disease Neuroimaging Initiative (ADNI) has demonstrated that biomarkers change over time in a predictable sequence [9]. This makes possible prevention studies looking at delaying biological progression over a relatively short time (12 to 18 months). For instance in early MCI (EMCI) there is already amyloid deposition but little PET-FDG changes and no atrophy on MRI [25]. Progression to late MCI (LMCI) will likely correlate with worsening of PET-FDG and early atrophy on serial MRI. This type of study would be considered as proof-of-concept and would be supportive for longer and larger clinical trials. Shorter (12 months) studies could even be done in APP or presenilin mutation carriers who are within 5 years of their expected time of dementia (ETD) based on their family history [16].

### Delaying cognitive decline

Delaying decline of cognition using a standardized cognitive measure may be a valid primary outcome in primary or secondary prevention studies [15]. The CogState appears to be of interest for epidemiological studies in older people [26] and in MCI [27] The episodic memory decline measured by the CogState correlates with findings on amyloid PET imaging [28], thus bridging cognition and biomarkers in pre-dementia stages of AD.

### Delaying dementia

The studies comparing Ginkgo Biloba in France [29] and in the USA [30] are good examples of randomized studies where time to dementia was the primary outcome. The low incidence rate of dementia caused the US study to be prolonged from the original five years to seven. Thus although having a high face validity, a delay of incident dementia may not be the ideal outcome because of the duration of studies and the need for a conversion committee on top of an experienced clinician opinion.

In the future, primary prevention trials of AD will still need to deal with the issues of enrollment of target population, safety of drugs, cost, and the length and outcome of follow up. Drugs chosen for testing in primary prevention trials should be demonstrated to be safe when administered to normal elderly [11]. Although time to diagnosis of dementia is at present still the primary outcome measure in the traditional prevention clinical trials of AD and there are no biomarkers replacing the clinical outcome of dementia or cognitive decline, biomarkers may assist or even become surrogate outcomes in the clinical trial of AD in the future [12].

## Clinical application of prevention

There is a need for a structured approach to the prevention of AD as new data becomes available. Groups of persons at different level of risk are already seeking advice from their family doctor and memory clinics. The baby boomers may flood the resources of specialized centers for AD who are currently responsible for the diagnosis and symptomatic treatment of AD and other dementias, and who will also have to deal with the use of disease-modifying drugs in the near future, some requiring monthly intravenous infusions. Hopefully family doctors with interest in prevention of heart disease and stroke will also be interested in AD prevention, since these conditions share many risk factors.

## Conclusions

The prevention of AD require large investment of time and money, but the return on investment may be huge, considering the projections of costs for patients with dementia in the near future. Regular meetings of clinical trialists and epidemiologists will facilitate the development of methodology for successful prevention studies, as was recently done under the auspices of the Alzheimer Association [31].

## Competing interests

The authors declare that they have no competing interest.

## Authors’ contributions

All authors contributing equally and having read and approved the manuscript.

## Author details

^1^McGill Center for studies in Aging, McGill University, Montreal, Canada.^2^Neurological Department, Xuanwu Hospital, Capital University, Beijing, China. ^3^McGill Centre for Studies in Aging (MCSA), McGill University, 6825, Boul. LaSalle Blvd, Montreal, QC H4H 1R3, Canada.
